# Supporting whānau during COVID-19 pandemic in Aotearoa New Zealand: a systems thinking case study

**DOI:** 10.1186/s12913-024-11164-z

**Published:** 2024-06-11

**Authors:** Sudesh Sharma, Cheryl Davies, Helena Rattray-Te Mana, Michael Baker, Amanda Kvalsvig, Mat Walton

**Affiliations:** 1 Institute of Environmental Science and Research, 27 Creyke Road, Ilam, Christchurch, 8041 New Zealand; 2 Kōkiri Marae Hauora and Te Rōpū Rangahau Hauora A Eru Pōmare, University of Otago, 7-9 Barnes Street, Seaview, Lower Hutt , Wellington 5010 New Zealand; 3 Institute of Environmental Science and Research, 34 Kenepuru Drive, Porirua, Wellington 5022 New Zealand; 4 University of Otago, 23A Mein Street, Newtown, Wellington 6021 New Zealand

**Keywords:** COVID-19, Equity, Systems thinking, Māori service provider, Whānau, Aotearoa New Zealand

## Abstract

**Background:**

The Aotearoa New Zealand COVID-19 pandemic response has been hailed as a success story, however, there are concerns about how equitable it has been. This study explored the experience of a collective of Māori health and social service providers in the greater Wellington region of Aotearoa New Zeland delivering COVID-19 responses.

**Methods:**

The study was a collaboration between a large urban Māori health and social service provider, Tākiri Mai Te Ata whānau ora collective, and public health researchers in Aotearoa New Zealand. Two online workshops were held with staff of the Māori service provider, collectively developing a qualitative causal loop diagram and generating systemic insights. The causal loop diagram showed interactions of various factors affecting COVID-19 response for supporting whānau (Māori family/households) at a community level. The iceberg model of systems thinking offered insights for action in understanding causal loop diagrams, emphasizing impactful changes at less visible levels.

**Results:**

Six interacting subsystems were identified within the causal loop diagram that highlighted the systemic barriers and opportunities for effective COVID-19 response to Māori whānau. The medical model of health service produces difficulties for delivering kaupapa Māori services. Along with pre-existing vulnerability and health system gaps, these difficulties increased the risk of negative impacts on Māori whānau as COVID-19 cases increased. The study highlighted a critical need to create equal power in health perspectives, reducing dominance of the individual-focused medical model for better support of whānau during future pandemics.

**Conclusions:**

The study provided insights on systemic traps, their interactions and delays contributing to a relatively less effective COVID-19 response for Māori whānau and offered insights for improvement. In the light of recent changes in the Aotearoa New Zealand health system, the findings emphasize the urgent need for structural reform to address power imbalances and establish kaupapa Māori approach and equity as a norm in service planning and delivery.

## Background

### Ehara taku toa i te toa takitahi, engari he toa taki tini

#### My strength is not that of an individual, but as a collective

The novel COVID-19 pandemic has impacted communities across the globe, requiring communities to draw upon their resources and strengths. In New Zealand or Aotearoa [Māori name for New Zealand (hereafter Aotearoa New Zealand)], Māori (Indigenous people of Aotearoa New Zealand) communities often bear the disproportionate socio-economic impact of such health issues which are deeply rooted in the legacy of colonisation including historical injustices and structural racism [1]. While the Ministry of Health has implemented some key policies and plans to advance Māori health [2, 3], inequities in health outcomes persist. The way Aotearoa New Zealand's health system dealt with COVID-19 is considered a success internationally [4], involving dynamic phases of lockdowns, restriction adjustments, and vaccination campaigns. However, the COVID-19 response was also a period of policy turbulence, including the re-surfacing of Māori health inequity [5, 6], reflecting the complex and evolving nature of the pandemic.

During the COVID-19 pandemic, Māori communities showed exceptional leadership in responding to the pandemic. Iwi (extended kinship group, tribe of Māori) and Māori health and service providers quickly organised ways to support whānau (primary family unit, extended family or family group of Māori society) and kaumatua (Māori elders, a person of status within the whānau) isolating at home. The providers drew from a values base, located within te ao Māori (Māori worldview) [7, 8]. Māori ways of working reflected ideas of collective action that are empowering and strength-based with unconditional manaakitanga (hospitality and kindness) to anyone in need [9]. Māori responses demonstrated self-reliance and localised self-determination [10].

While drawing upon various resources, community efforts were also initially supported by the government through flexibility in existing contracts, and later with several rounds of additional funding directly to Māori providers [11, 12]. Cultural practices were adapted to keep kaumatua and vulnerable whānau safe. For example, practices at tangihanga (funeral) were adapted in line with government guides to reduce infection rates [10]. Iwi were also able to accelerate the slow start coverage of vaccination for Māori by adapting the non-Māori interventions to kaupapa Māori (Māori approach, customary practice or ideology) based responses [8, 9]. Māori service providers played a key role in delivering culturally authentic responses that made sense to whānau and were effective in many ways [7–10].

Despite these successes, there was concern that existing health access-related inequities and associated health outcomes would be repeated in relation to COVID-19 response [4]. Whilst total population impacts of the COVID-19 elimination strategy have been positive [13], evidence has emerged that whānau Māori have been disproportionately impacted. Choi and colleagues (2021) reported Māori whānau with low incomes faced significant socio-economic and psychological challenges due to COVID-19, even though they were quite positive about elimination measures [14]. Further, whānau Māori experienced poorer access to information, higher hospitalisation rates, and lower and slower vaccination rates [15]. Most importantly, Māori and Pacific communities experienced a higher risk of hospitalisation and mortality due to COVID-19 [5, 6].

The government response has been criticised for not sufficiently regarding Māori advice early in the pandemic response, a requirement under the Treaty of Waitangi [16, 17]. The Treaty of Waitangi was signed by the British Crown and Māori Chiefs in 1840 to establish British governance in Aotearoa New Zealand. It embodies the principles of partnership, self-determination, and equity between Māori and the Crown, representing the non-Māori communities. Systemic barriers to kaupapa Māori based responses were evident during the early phases. For instance, in one South Auckland clinic, the standard government response for supporting whānau isolating at home did not meet whānau needs, with additional whānau-centred support provided [18]. The whānau-centred services ensured that whānau received holistic support in accordance with te ao Māori in a way that was equitable and upheld the Treaty of Waitangi commitment.

Systems thinking and complexity science-based approaches can help illustrate such complex problems, and indicate barriers and opportunities for contextual responses [4, 19]. Such approaches have a range of applications including enhancing theory of change, illustrating complexity, engaging with stakeholders, evaluating programs, and more [4, 19–24]. In the context of COVID-19, systems methods and tools enable a collaborative understanding of social determinants and the impact of COVID-19 pandemic. Systems thinking approaches fit the complex nature of public health problems and aim to support deeper understanding and learning within public health research and practice.

Systems thinking tells us that patterns are often stable over time and hard to shift. The colonial structures of health and social services continue to dictate the distribution of resources and information in a Euro-centric way despite efforts to adhere to Māori-led approaches in Aotearoa context. Given the historical marginalisation of Māori-defined and Māori-led approaches within the health system, and persistent inequities in access to healthcare services and health outcomes for Māori, a reproduction of inequitable outcomes was a risk in COVID-19 response. Conceptually, this pattern was shown in a scoping review of social determinants of COVID-19 response and recovery in the context of Aotearoa New Zealand [4].

This research sought to use systems thinking tools to look at the experience of Tākiri Mai Te Ata Whānau Ora Collective (henceforth, Tākiri Mai), delivering kaupapa Māori-led responses in supporting whānau through COVID-19. The study aimed to develop a systems thinking informed understanding of challenges for services and whānau related to the COVID-19 pandemic and consider opportunities for improving whānau support during future COVID-19 waves or other pandemics. The whānau was the unit of analysis to align with Māori cultural values, emphasizing collective well-being over individualism, in contrast to the Eurocentric perspective.

### Description of Tākiri Mai Te Ata whānau ora collective and its approach during COVID-19

Tākiri Mai is a collective of Māori Service providers including Kōkiri Marae Keriana Olsen Trust that delivers whānau-centred services that promote wellbeing and development. They provide a range of health and social services, including primary health services, outreach diabetes services, parenting programmes, school holiday programmes, and breast screening to primarily Māori communities in the greater Wellington region of Aotearoa New Zealand. Tākiri Mai is guided by Māori worldviews encompassed within a values framework, to provide mana-enhancing (prestige enhancing, empowering) and whānau-centric services to anyone in need. These values were key to the COVID-19 response by Tākiri Mai services since March 2020 [9]. ‘Kaitiakitanga’ (stewardship) and ‘manaakitanga’ (hospitality and kindness) were key mantras for whānau responses by Tākiri Mai, from public COVID-19 prevention messaging to supporting daily essentials, to providing health services (COVID-19 testing and vaccinations). Tākiri Mai endeavoured to provide whānau-centred COVID responses that were unconditional (for example, delivering as many food parcels at the doorsteps of whānau without asking any questions) and mana-enhancing (for example, making whānau comfortable and providing services respectfully and kindly without any judgment), despite constraints presented by how the wider system was organised.

## Methods

### Study design

The Institute of Environmental Science and Research in partnership with Kōkiri Marae Keriana Olsen Trust, Tākiri Mai Te Ata Whānau Ora Collective and the University of Otago conducted this collaborative case study. The case study methodology enabled an in-depth exploration of the experience of Māori service providers, employing qualitative techniques such as focus group discussion, group model building and sense making of the model using the iceberg model of systems thinking [25].

Group model building is a participatory approach that allows for the integration of different perspectives, knowledge and evidence in the form of systems diagrams, in this case, an influence diagram and a causal loop diagram [26, 27]. Causal loop diagrams are at a higher level of abstraction than influence diagrams and both were presented back to participants to check the link between what was discussed within the workshop and the causal loop diagram. A typical causal loop diagram has four key elements: variables (e.g. COVID-19 community cases), causal links (indicated by arrows with ± sign), delays (indicated by two short parallel lines on the links) and feedback loops (closed loops formed by two or more causal links). A positive causal link indicates influence or change in the same direction (e.g. with an increase in socio-economic vulnerability, the risk of COVID-19 infection will also increase). A negative link indicates change in the opposite direction (e.g. with an increase in whanau-centred COVID-19 response, the risk of COVID-19 infection among Māori communities will decrease). Further, there are two types of feedback loops, balancing and reinforcing loops. Balancing loops seek to stabilise the system and maintain the status quo, and therefore tend to create resistance for further change in each direction (e.g. increase in health sector interventions (such as vaccination and isolation) lead to decrease in COVID-19 infections, which then lead to decrease in health sector interventions). Reinforcing loops produce an ever-increasing or decreasing change leading to rapid growth or decline (e.g. a vicious cycle of poverty and poor health status). We have also used the concept of system archetypes, simpler versions of causal loop diagram that illustrate some common behaviour of systems to discuss systemic patterns emerging from our study [28].

The iceberg model of systems thinking helped make sense of the causal loop diagram by bringing in deeper insights about leverage for action [29, 30]. The iceberg model is similar to Donella Meadows’ concepts of system leverage points [31, 32] and both models were used to make sense of the causal loop diagram. The key idea of the iceberg model is that greatest change is achieved through changes at lower levels of the iceberg that are not immediately visible. The greatest change can occur through a shift in mental models – how people view and think about the world, from which decisions and actions arise. System structures (e.g. policies, funding practices), which are influenced by mental models, are the next most effective location for change. System structures set patterns that we can observe, such as persistent health inequities. We can intervene directly on these patterns, but our interventions may be less effective at change, as the underlying drivers of the patterns are system structures and mental models. Finally, particular events (e.g. individuals with disease) are the easiest place to intervene but do little to change patterns and underlying system structures.

The study built on the scoping literature review undertaken by the research team which highlighted the gaps in the Aotearoa COVID-19 response and, as a result, potential inequitable outcomes [4]. For this study, two workshops (120 min each) were held with managers across Tākiri Mai to develop (workshop one) and refine (workshop two) a causal loop diagram through group model building processes. Conducting the workshops online was a practical necessity due to the Omicron outbreak in Aotearoa during the planned workshop dates. Both workshops were adapted for an online setting, which meant the development of the causal loop diagram occurred outside of the workshop, using information gathered in workshop one. Six participants were recruited through internal discussions within Tākiri Mai. All participants of the workshop received details regarding the study, provided their informed written consent to take part, and consented to have their information recorded and utilised for analysis. The study received ethics approval from the University of Otago Human Ethics Committee (Ref. no. D21/425) and was conducted in strict adherence to the ethics approval.

### Data collection, analysis and refinement

We conducted the first workshop via video conference, consisting of a focus group discussion among six participants. Notes of the discussion were captured in real time on Miro interactive whiteboard, so that notes could be seen within the workshop, and would also be available to participants anytime. A connection circle activity was completed to link observations [27]. The connection circle is a tool that helps to identify relationships and feedback among different elements of a system, in this case, the notes on the Miro board.

The data from the first workshop was used to develop an influence diagram and a causal loop diagram embedded within an iceberg model. The influence diagram incorporated what was discussed during the first workshop and mapped chains of influences among the issues discussed. The influence diagram informed the development of the causal loop diagram. The causal loop diagram extended the influence map by illustrating the patterns of interaction.

The causal loop diagram focused on dynamics between social and economic determinants of health, public health responses, recovery interventions and impacts of economic recession, and recovery of health and wellbeing. The initial model was developed and thoroughly checked by all authors to represent the views shared during the first workshop. The draft model was then shared with participants through Kumu, a collaborative systems mapping software [33]. The second workshop and subsequent online engagement focused on refinement and “making sense” of the causal loop model with six participants from the first workshop. Specifically, the second workshop refined and validated the causal loop diagram from participant perspectives as a representation of the situation experienced in supporting whānau during the COVID-19 pandemic, as well as identifying areas where changes could be made to improve whānau outcomes.

## Results

Figure one shows a causal loop diagram of factors involved in supporting whānau during COVID-19, that were identified by managers and service providers of Tākiri Mai. The causal loop diagram is a visual representation of processes, issues and factors that interact and interconnect, consequently highlighting subsystems and loops.

**Fig. 1:**
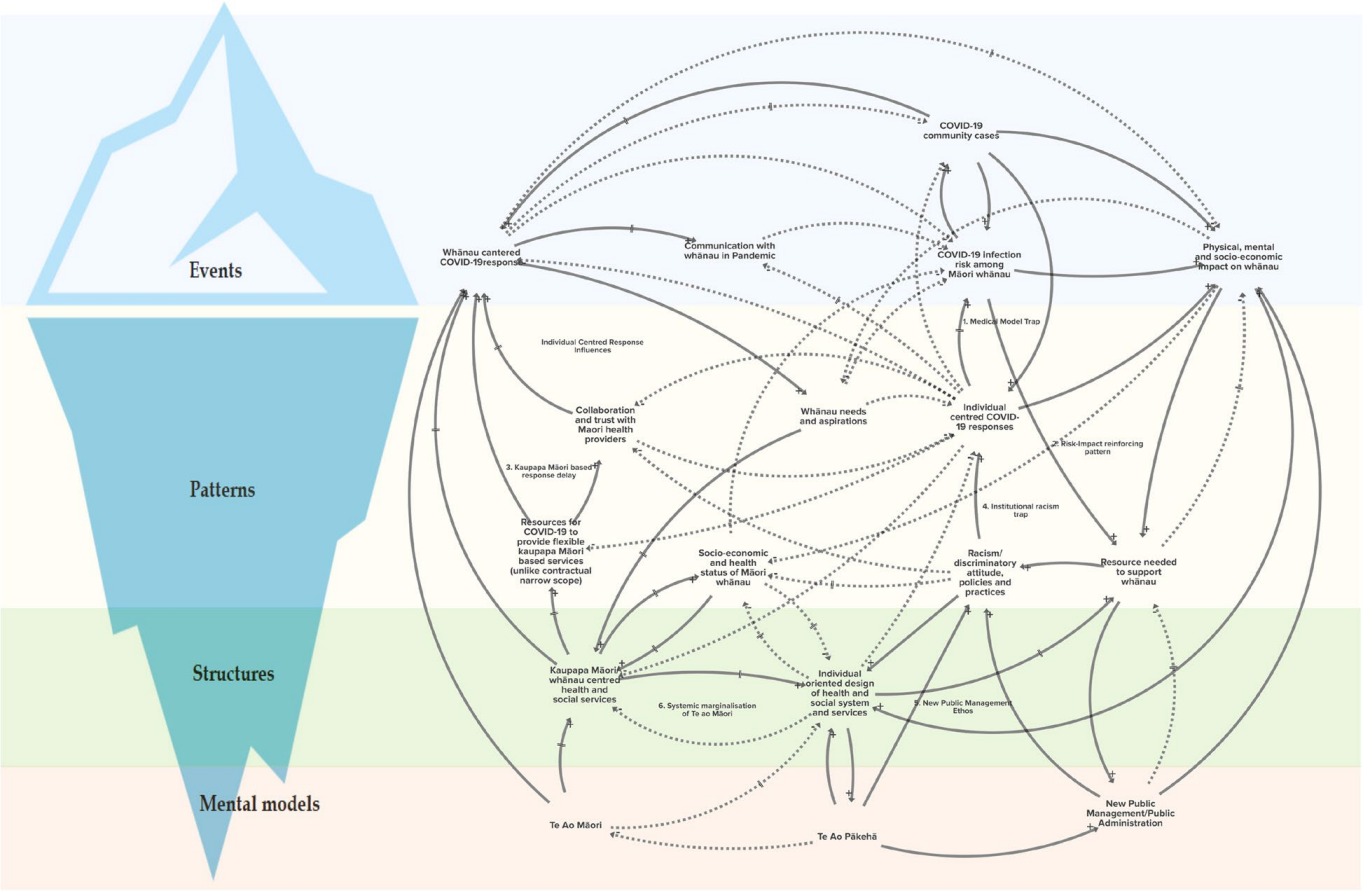
Iceberg model of systems thinking embedded causal loop diagram showing interactions among factors relating to COVID-19 support to whānau (see in-text details about key elements of causal loop diagram including linking arrows, signs, delays and loops)

Six subsystems within the causal loop diagram (Fig. 1) were identified that described the experience of participants in workshops and their perspective of factors contributing to those experiences.

### Subsystem 1: The medical model hinders COVID-19 related kaupapa Māori approach and services to whānau

While the overall COVID-19 elimination strategy during 2020/21 had benefits at a whole population level, and potentially pro-equity benefits, practically many actions at the community level were delivered by health services that are dominated by individual-focused medical models. Participants suggested that for Māori whānau, services tended to reduce the use of kaupapa Māori and strength-based approaches to wellbeing. The medical model operates as a balancing loop that acts to keep the overall system focused on individuals, slowing down opportunities to grow whānau-centred services. The reinforcing sub-system 1 shown in Fig. 1 operates at the patterns level of the iceberg and suggests that as COVID-19 cases grow, the pressure for an individual service response is reinforced.

### Subsystem 2: Risk-impact reinforcing pattern increases disproportionate impact to whānau

The risk-impact subsystem 2 operating at the patterns level (see Fig. 1) suggests that as COVID-19 cases increase, negative impacts on whānau were likely to increase. The starting position of whānau in terms of social and economic determinants of health were shown – that fewer social and economic resources were likely to increase COVID-19 risk, and increase negative impacts on whānau wellbeing. The subsystem dynamics showed the impact of increasing COVID-19 cases on the ability of Tākiri Mai to deliver support services to whānau, as staff became sick and isolated themselves. As a response, some agencies attempted to deliver more services online or by phone, which only suited the needs of some whānau. The risk-impact reinforcing pattern suggests longer-term change requires action on improving whānau access to social and economic resources. Shorter-term responses may focus on maintaining or enhancing service response from agencies in times of high community cases.

### Subsystem 3: Kaupapa Māori-based, whānau-centred and strength-based approaches are effective but delayed

The subsystem 3 which also operates at the patterns level (see Fig. 1) shows that ultimately, whānau-centred services have potential to improve outcomes for whānau. However, individual-focused services still dominated and delayed whānau-centred services. Participants reported slow COVID-19 responses for supporting whānau and that communication with whānau had been difficult or ineffective, indicating the dominance of individual and diseas-focused responses. Participants also described slower and lower than ideal levels of collaboration and trust between funders and other providers in the area. While whānau-centred and whānau ora services were available and operating they had not been enabled to undertake significantly more of the service response for whānau in a systematic and well-resourced way.

### Subsystem 4: Institutional racism limits whānau-centred approach

The subsystem 4 operating at the patterns level (see Fig. 1) can be viewed alongside the whānau-centred approach delay and medical model trap, all acting to limit the expansion of whānau-centred approaches. Participants considered institutional racism as underpinning some relationships and communication between agencies, as well as leadership style and emphasis of individuals compared to whānau-centred services. A privileging of te ao Pākehā (western worldview in the context of Aotearoa New Zealand) overrides te ao Māori as overarching paradigms informing service response. This sub-system includes elements across all levels of the iceberg model and indicates systemic challenges.

### Subsystem 5: Neoliberalism marginalises whānau-centred responses

The subsystem 5 operating at the structures level of the ice-berg (see Fig. 1) shows the reinforcing effect of neoliberalism to maintain individual service responses as a dominant pattern, which limited the breadth of whānau-centred responses. Participants discussed how some contracts were structured in a way that limited whānau-centred responses to COVID-19. For example, participants felt leadership from some agencies focused on services, rather than meeting the needs of whānau and thereby acted to restrict resource distribution. It indicated the paradigm of new public management, which underpinned much of service contracting in Aotearoa, reflecting a dominant te ao Pākehā paradigm.

### Subsystem 6: Te Ao Māori is systemically marginalised across health system design and functioning

The subsystem 6 operating at the structures level (see Fig. 1) depicts a situation where te ao Māori is systematically marginalised, keeping kaupapa Māori service development more generally at the periphery, which in turn keeps whānau-centred COVID-19 responses as marginal. This sub-system suggested initial dominance of te ao Pākehā was maintained because of delays in enabling a te ao Māori approach and experience of kaupapa Māori services on the majority of service design and funding decisions.

### Opportunities for action

**Fig. 2:**
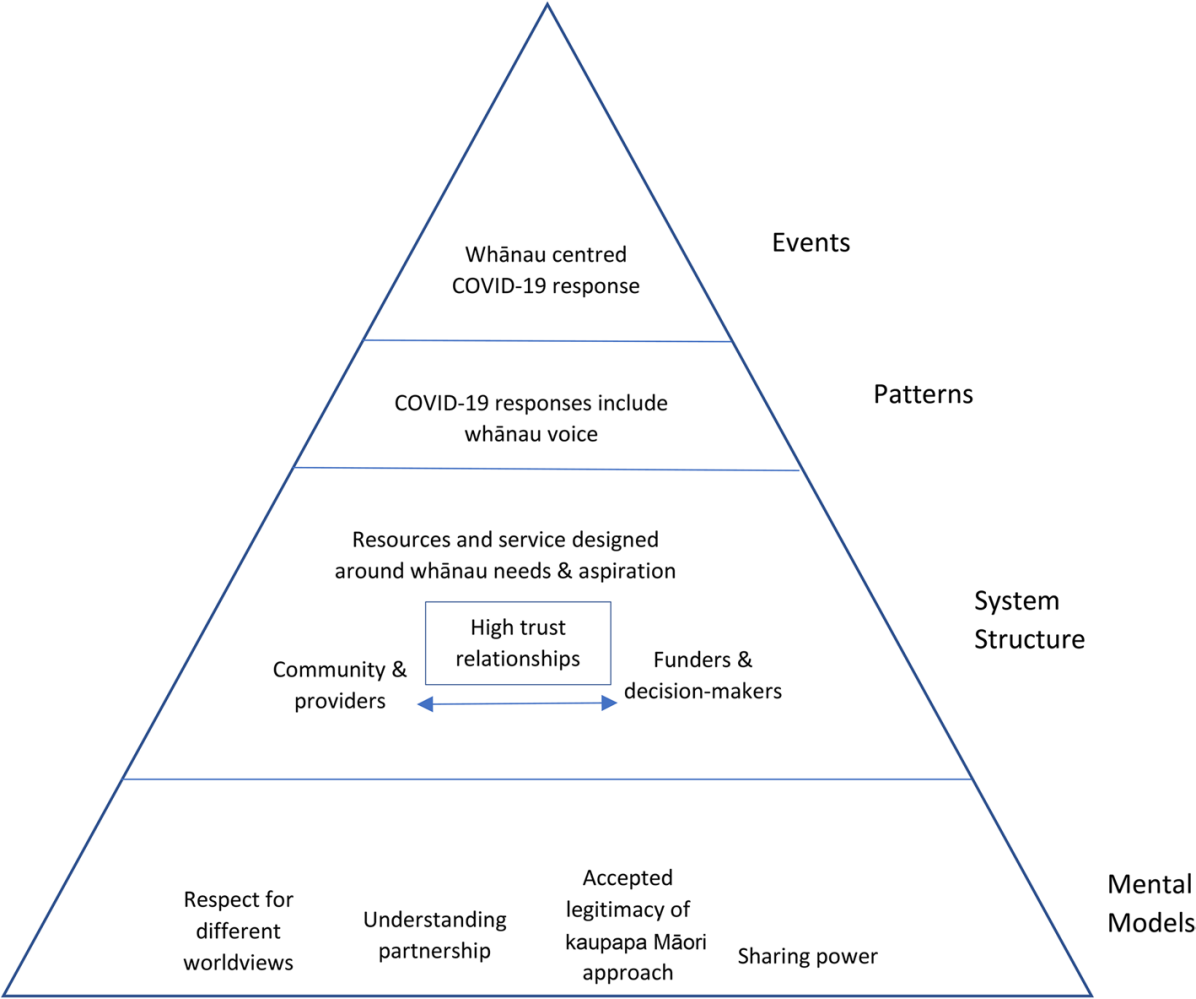
Systems leverage points for improving whānau-centred COVID-19 response based on the causal loop diagram (Fig. 1)

In the second workshop and subsequent online engagements, participants considered opportunities for action in relation to the system insights within the causal loop diagram, with a focus on how whānau can be better supported in future COVID-19 outbreaks or other pandemics. Drawing upon the iceberg model and Donella Meadows’ concepts of system leverage points [31, 32], Fig. 2 illustrates that the most fundamental action rests on creating equal power between perspectives on health, which means reducing dominance of the individual focused medical model.

This required a shift towards mental models such as respecting different worldviews, accepting legitimacy of kaupapa Māori approaches including mātauranga (knowledge) Māori and sharing of power (bottom level of Fig. 2). Shifting mental models is likely the most effective place for change, but also likely to take the most time. Action on respect and understanding for worldviews and kaupapa Māori led approaches, and willingness to share power will likely support an increase in high trust relationships.

Mātauranga Māori inclusiveness would ensure Māori are involved in the decision-making process from the start of the COVID-19 response. In turn, high trust relationships and structural changes will support the services and resources designed around whānau aspirations and needs. The approach of government COVID response changed from elimination strategy [34] to suppression [35] and then a mitigation/management approach [36]. It could be argued that active protection of the whole population would be more effective if the strategy used a tight mitigation approach as this could also protect Māori whānau. Very low levels of disease circulating within the community would mean that issues identified in subsystem 2 would become less evident, creating better opportunities for change.

High trust relationships and equal power of worldviews are likely to support new ways of service design, contracting and implementation, increasing whānau-centred approaches. The new way of designing services involves moving towards flexible funding and contracting methods that back a kaupapa Māori approach and a whānau-centered approach. The focus is on enabling a more adaptable and culturally attuned framework that aligns with kaupapa Māori principles and acknowledges the importance of whānau wellbeing, prioritizing it over inflexible, resource-constrained Western models. From these system structure changes, a pattern of positioning whānau voice as central in how services are planned and delivered would develop. These changes at the mental model, system structure, and pattern levels are likely to see an increased number of whānau-centred services, even when services are, by necessity, reactionary in responding to a pandemic situation. In a situation where mental models and high trust relationships are not already established prior to a pandemic situation, whānau-centred services can likely be established through processes that purposefully incorporate Māori service providers early in planning, which will also support longer-term relationship building and influence mental models.

## Discussion

The study aimed to understand systemic barriers and opportunities in supporting whānau through COVID-19 from a Māori health and social service provider perspective. The study developed a causal loop diagram to illustrate those key systemic barriers and opportunities to support whānau. As a single case study, no claim is made that the findings represent the experiences of other Māori health and social service providers. However, the systems insights developed from this one case study can be used as a lens to explore the experience of other providers and ways in which government COVID-19 responses are designed.

It is also acknowledged that numerous successes of Māori led COVID-19 responses have been about Iwi and Māori providers exercising rangatiratanga (right to exercise authority), and getting on with responses without waiting for the government [9, 10]. Further, the study did not follow an ideal group model building approach to develop the causal loop diagram. The group model building was adapted for online workshop settings and developed by the study team in Kumu for sharing and seeking feedback on the causal loop diagram from participants asynchronously. The second workshop ensured participants validated the causal loop diagram and identified opportunities for action.

The causal loop diagram depicted complex interactions of systemic traps and delays relating to the COVID-19 pandemic response. Many whānau faced a challenging situation with COVID-19 directly [5, 6], and less directly due to COVID-19 impacts on relationships, income and access to multiple services [4, 37–39]. There are numerous examples of Iwi and Māori health and social service agencies taking action to support whānau during COVID-19, drawing upon tikanga (customary system of values and practices of Māori society) and displaying local rangatiratanga [7–10]. The government has actively considered and resourced Māori-led responses to COVID-19 [40] yet the majority of COVID-19 responses were not designed from the perspective of Māori tikanga and worldviews, instead reflecting individual and medical model focused approaches. Many Māori whānau are likely to be interacting with services designed for the general population.

Through this case study of a single urban Māori health and social service provider, it was clear that tension existed at the boundaries of whānau-centred approaches within dominant non-Māori approaches. The participants’ experience was of kaupapa Māori providers being peripheral in the design of services, and more often left to “clean up” messes than be involved in planning for good whānau outcomes at the outset, an experience reported elsewhere [15, 16]. Planning, funding and contracting processes are designed for majority services informed by “Western” concepts of health and public administration. This general situation caused delays in whānau-centred COVID-19 responses, keeping kaupapa Māori-led and whānau-centred responses marginal within the overall COVID-19 situation.

**Fig. 3:**
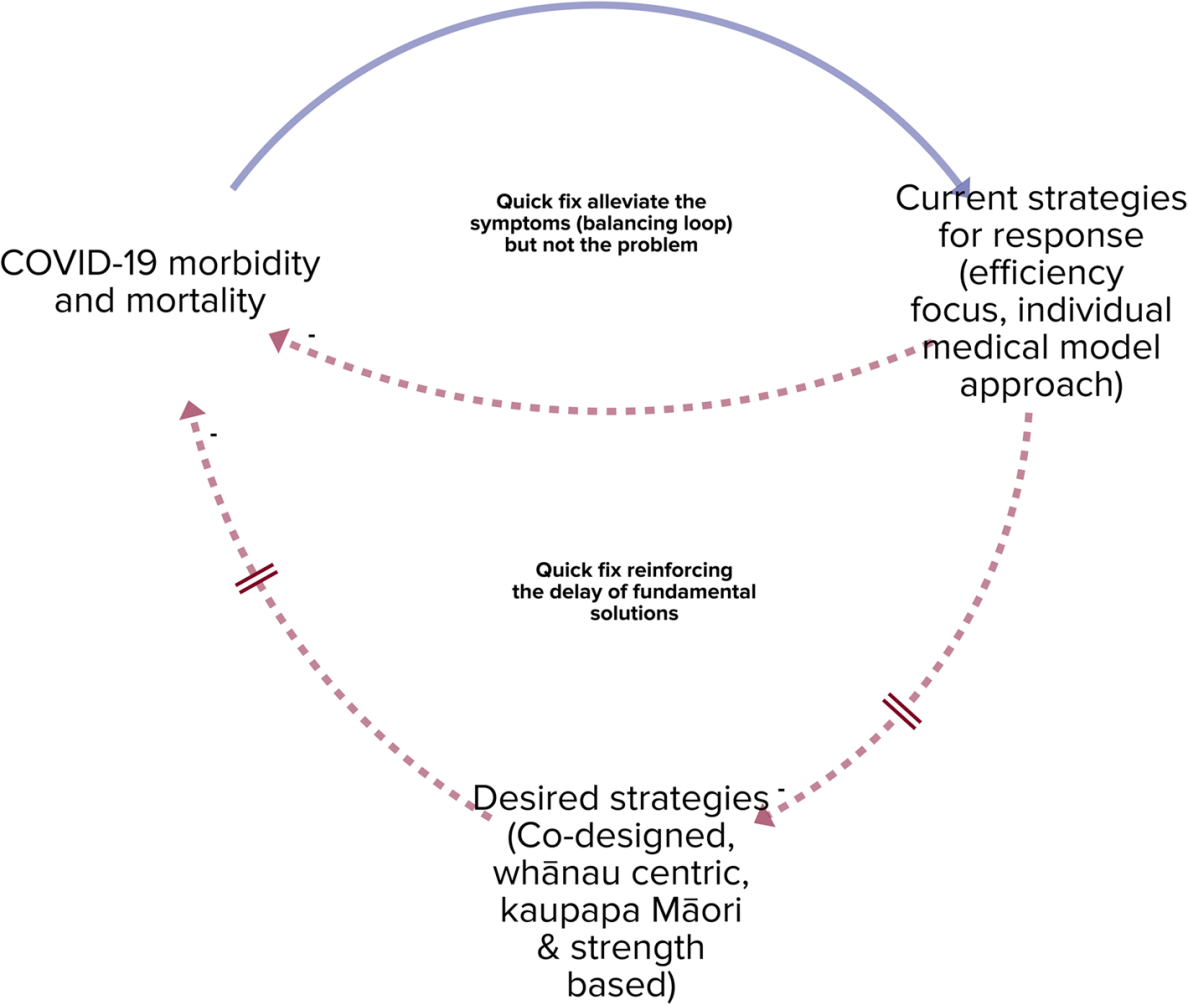
‘Fixes that fail’ systems archetype showing interaction of short-term and long-term COVID-19 responses for whānau-centred services

The situation is simplified in Fig. 3. In systems thinking language, this situation is known as ‘fixes that fail’, where short-term solutions erode action on long-term solutions [28]. Within a context of current health reforms and intended outcomes to support Pae Ora (Healthy Futures) as stated within *Whakamaua: Māori Health Action Plan 2020–2025*, enabling whānau-centred kaupapa Māori-based health responses is a goal that should be more evident within COVID-19 responses [6].

Underpinning a focus on short-term responses identified in Fig. 3, is the dominant place of the individual-focused medical model of health interventions. The individual medical model worldview reduces the opportunity for a whānau-centred worldview coming from te ao Māori. The individual medical model aligns more easily with new public management approaches to service design, contracting and management. The clash of new public management and Māori-led services has been identified through numerous studies [41–44]. While a whānau-centred service may usefully reduce morbidity and social impact of COVID-19, the dominance of individual and medical-focused responses limit ability to grow whānau-centred approaches. Dominance of the individual focused medical model may also crowd out population health approaches, where responses focus more on reducing rates of disease across populations, rather than treatment of individuals.

The government has been criticised for lack of early engagement with Māori for responding to the COVID-19 pandemic [15, 16], building upon historical evidence of inequitable access to health services [45]. The COVID-19 response was led by a health system transitioning to a way of working that places Māori perspectives more prominently within policy and delivery [3]. The COVID-19 response showed that rapid service design and response is possible. It also showed the difficulty of rapid response that addresses, rather than repeats, inequities built into the health system over a long time period [17].

The experience of Tākiri Mai Whānau Ora Collective, shows responses to support whānau during the COVID-19 pandemic continue to reinforce ineffective ways of working within the health system and wider government systems with communities. The impact of continued structural inequities is well recognised, with a clear intention of creating change through the health system reforms [2, 3]. The establishment of the Māori Health Authority (Te Aka Whai Ora) could be considered as a key structural reform to influence the mental models and system structures towards equitable outcomes for Māori based on whānau-centred responses and high trust relationships [46]. However, a change in government in late 2023 has led to the decision to disestablish the Māori Health Authority as a stand-alone entity and instead integrate its functions within the Ministry of Health [47]. This situation underscores the influence of political dynamics in impeding systemic reforms at the highest levels, illustrating how changes in government can impact crucial initiatives designed to address health inequalities.The disestablishment poses a setback to the systemic intervention that was intended to reshape prevailing mental models and narratives, hindering progress towards health equity.

Pandemic responses are built upon existing health system capacities, capabilities, and processes. Recent literature recommends pro-sociality framing for future pandemic responses across the system in order to break the medical model trap, incorporating collective action and social cohesiveness, and making responses (and underlying decision making) equity focused across the health system [48]. In the evolving health landscape of Aotearoa New Zealand with an enhanced focus on equity and pae ora, there is potential for future pandemic responses to support and grow whānau-centred and pro-sociality approaches. There is an opportunity to not only address the immediate challenges posed by pandemics but also to enhance overall community well-being. This proactive shift can contribute to a more resilient and interconnected health system that prioritizes equity, collective well-being, and Māori-led responses in the face of future health challenges.

## Conclusions

The study used a systems thinking approach to understand the COVID-19 all-of-government response in Aotearoa through the perspective of a Māori health and social service provider. The study provides insights on systemic traps, their interactions and delays contributing to a relatively less effective COVID-19 response for Māori whānau. It also demonstrated the possibility and value of whānau-centred services, despite a reactionary mode of operation during emergencies. The study supported the need for transformative shifts in worldviews driving health systems planning and action. The importance of engaging with Māori service providers early in a pandemic situation, based on respectful and equitable partnership relationships, is critical for equitable outcomes. With the recent disestablishment of the Māori Health Authority, the necessity for structural reform is now more crucial than ever, as it holds the potential to initiate a paradigm shift in narratives, address power imbalances at the highest level, and actively embed kaupapa Māori approach and equity as the norm in service planning and delivery. The study's findings also hold significance for countries with comparable colonial histories, providing insights into mitigating health inequalities and cultivating partnerships with Indigenous communities. The emphasis on the kaupapa Māori approach and structural reform offers a transferrable framework for reclaiming cultural agency in the health system within similar colonial contexts.

## Data Availability

The dataset that is anonymised and deidentified will be made available from the corresponding author on reasonable request.

## Glossary of Māori terms

Aotearoa: Māori name for New Zealand
Hauora: Health, vigour
Iwi: Extended kinship group, tribe of Māori
Kaitiakitanga: Guardianship, stewardship
Kaumātua: Māori elders, a person of status within the whānau
Kaupapa Maori: Māori approach, customary practice or ideology
Māori: Indigenous people of Aotearoa New Zealand
Mātauranga Māori: Māori knowledge based on indigenous wisdom, broadly include concepts, philosophies and understanding based on Māori worldview
Mana: Prestige, influence, power
Manaakitanga: Hospitality, kindness, generosity, support - the process of showing respect, generosity and care for others
Marae: Communal sacred meeting place or space for Māori communities where formal greetings and discussions take place
Pae Ora: Healthy Futures
Rangatiratanga: Right to exercise authority, chiefly autonomy, chiefly authority originating from Māori worldview
Tangihanga: Funeral
Te Aka Whai Ora: Māori Health Authority
Te Ao Māori: Māori worldview
Te Ao Pākeha: European settlers/western worldview in the context of Aotearoa
Tikanga: Customary system of values and practices of Māori society
Tino rangatiratanga: Self-determination
Whānau: Primary family unit, extended family or family group of Māori society
Whānau ora: Health and vitality of family/extended family
